# A Versatile Flow Reactor Platform for Machine Learning Guided RAFT Synthesis, Amidation of Poly(Pentafluorophenyl Acrylate)

**DOI:** 10.1002/marc.202500264

**Published:** 2025-04-08

**Authors:** Alexander P. Grimm, Stephen T. Knox, Clarissa Y. P. Wilding, Harry A. Jones, Björn Schmidt, Olga Piskljonow, Dominik Voll, Christian W. Schmitt, Nicholas J. Warren, Patrick Théato

**Affiliations:** ^1^ Institute for Biological Interfaces III (IBG‐3) Soft Matter Synthesis Laboratory Karlsruhe Institute of Technology (KIT) Hermann‐von‐Helmholtz‐Platz 1 76344 Eggenstein‐Leopoldshafen Germany; ^2^ School of Chemical and Process Engineering (SCAPE) University of Leeds (UoL) Woodhouse Leeds LS2 9JT UK; ^3^ School of Chemical Materials and Biological Engineering University of Sheffield (UoS) Mappin Street Sheffield S1 3JD UK; ^4^ Institute for Technical Chemistry and Polymer Chemistry (ITCP) Karlsruhe Institute of Technology (KIT) Engesserstraße 18 76131 Karlsruhe Germany

**Keywords:** digital chemistry, flow chemistry, post‐polymerization modification, RAFT‐polymerization, self‐optimization

## Abstract

Data‐driven polymer research has experienced a dramatic upswing in recent years owing to the emergence of artificial intelligence (AI) alongside automated laboratory synthesis. However, the chemical complexity of polymers employed in automated synthesis still lacks in terms of defined functionality to meet the need of next‐generation high‐performance polymer materials. In this work, the automated self‐optimization of the reversible addition‐fragmentation chain‐transfer (RAFT) polymerization of pentafluorophenyl acrylate (PFPA) is presented, a versatile polymer building‐block enabling efficient post‐polymerization modifications (PPM). The polymerization system consisted of a computer‐operated flow reactor with orthogonal analytics comprising an inline benchtop nuclear magnetic resonance (NMR) spectrometer, and an online size exclusion chromatography (SEC). This setup enabled the automatic determination of optimal polymerization conditions by implementation of a multi‐objective Bayesian self‐optimization algorithm. The obtained poly(PFPA) is precisely modified by amidation taking advantage of the active pentafluorophenyl (PFP) ester. By controlling the feed ratios of solutions containing different amines, their incorporation ratio into the polymer, and therefore its resulting properties, can be tuned and predicted, which is shown using NMR, differential scanning calorimetry (DSC), and infrared (IR) analysis. The described strategy represents a versatile method to synthesize and modify reactive polymers in continuous flow, expanding the range of functional polymer materials accessible by continuous, high‐throughput synthesis.

## Graphical abstract

The automated optimization of the polymerization of pentafluorophenyl acrylate in continuous flow is reported. Optimal reaction parameters suggested by an optimization algorithm are validated by an independent flow reactor setup. The control over modification of poly(PFPA) with primary amines is showcased by ^19^F NMR, ^1^H NMR, and IR spectroscopy techniques.

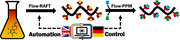

## 1 Introduction

With the emergence of AI and autonomous laboratory management, chemical synthesis has experienced significant advancements in terms of high‐throughput data collection and interpretation. From small organic molecules,^[^ ^1^ ^]^ inorganic materials,^[^ ^2^ ^]^ high‐entropy alloys,^[^ ^3^ ^]^ and biomedical drugs^[^ ^4^ ^]^ to next‐generation energy storage systems,^[^ ^5^ ^]^ AI‐supported synthesis has entered every field of chemistry. The availability of real‐time data analysis facilitates efficient reaction optimization and material composition investigation through rapid screening in a high‐throughput manner. Additionally, a continuous reaction platform can be adapted into an autonomous system, requiring only an intelligent algorithm to implement an iterative optimization process. Such an algorithm can then, for example, analyze the real‐time data and control the reaction by adjusting the concentrations of reactants and the flow rates of the starting materials.^[^ ^6^ ^]^ For this, flow processing provides considerably more stable and consistent reaction conditions than batch processing. This enhanced stability and reproducibility arise from the larger surface area‐to‐volume ratio, leading to isothermal conditions, efficient heat dissipation, and straightforward scalability.^[^ ^7^ ^]^ Furthermore, the continuous nature of flow processes makes them excellent candidates for online monitoring techniques, as the progress of the reaction can directly be tracked by analyzing the output stream.^[^ ^8^ ^]^ Importantly, the fields of polymer chemistry and supramolecular chemistry were greatly advanced in recent years by the control over reaction conditions and the ability to rapidly screen different chemical systems for optimizing polymerization reactions and rapid scale‐up, resulting in enhanced reaction consistency, improved safety, and increased sustainability.^[^ ^9^ ^]^ By leveraging large datasets and sophisticated algorithms, machine learning can predict properties, optimize reaction conditions, and facilitate the design of new polymer materials.^[^ ^10^ ^]^ Synthetic polymer chemistry often faces a challenging balance between process efficiency and product quality. A key factor in obtaining controlled polymer properties, such as molecular weight and molar mass dispersity (*Đ*), is the use of reversible deactivation radical polymerization techniques. Among these techniques, reversible addition‐fragmentation chain transfer (RAFT) polymerization stands out due to its robustness and ease of application.^[^ ^11^ ^]^ Efficiently balancing the trade‐off between reaction speed and product quality to optimize polymerization conditions presents a significant challenge of current polymer research. By systematically exploring the relationship between conversion rates and *Đ*, researchers can identify the optimal balance of control achievable within specific condition limits. This approach could significantly enhance the efficiency and quality of polymer synthesis, making RAFT polymerization a more effective tool in synthetic polymer chemistry.^[^ ^12^ ^]^ In recent years, the traditional RAFT polymerization has been greatly advanced by the invention of photo‐iniferter RAFT (PI‐RAFT),^[^ ^13^ ^]^ electro‐RAFT (eRAFT),^[^ ^14^ ^]^ sono‐RAFT,^[^ ^15^ ^]^ polymerization‐induced self‐assembly (PISA),^[^ ^16^ ^]^ single unit monomer‐insertion (SUMI),^[^ ^17^ ^]^ and automated high‐throughput RAFT polymerization techniques.^[^ ^18^ ^]^ As a tool, arguably as important as controlled polymerization itself, post‐polymerization modification (PPM) has been growing in relevance in recent years.^[^ ^19^ ^]^ PPM allows for the synthesis of intricate polymers by modifying pendant reactive groups on macromolecules after polymerization. This method enables the easy and systematic production of (co)polymers that cannot be obtained through direct polymerization, as well as the precise adjustment of polymer composition, hence resulting in material properties that differ from those of the original polymers.^[^ ^20^ ^]^ Among the many modification strategies available to polymer chemists, the amidation or transesterification of active ester containing polymers has proven to be one of the most efficient methods, with pentafluorophenyl (meth)acrylates (PFP(M)A) being their most prominent parent building blocks alongside *N*‐hydroxysuccinimide (meth)acrylate (NHS(M)A) whose amidation runs quantitatively on the minute scale.^[^ ^21^ ^]^ While NHS‐based active ester homopolymers are known to suffer from limited solubility and faster hydrolysis,^[^ ^22^ ^]^ the applicability of pentafluorophenyl esters as precursor polymers for sophisticated chemistry ranges from unique polymer architectures,^[^ ^23^ ^]^ over metal‐catalyzed small molecule synthesis,^[^ ^24^ ^]^ to advanced data storage and cryptography materials,^[^ ^25^ ^]^ showcasing the versatility of PPM of active esters toward next‐generation polymer materials. Thus, investigation of poly(PFPA) as precursor polymer for functional materials by virtue of flow chemistry represents an exciting opportunity toward complex polymer architectures at unprecedented speed, control, and efficiency. The use of ^19^F NMR in combination with machine‐learning‐assisted polymer synthesis was reported in 2021 by Reis et al., who demonstrated the development of fluorinated copolymers for ^19^F magnetic resonance imaging agents via AI.^[^ ^26^ ^]^ Despite the 100% abundance of ^19^F, the high gyromagnetic ratio, and its larger chemical shift range, ^19^F NMR techniques remain underrepresented in flow chemistry.^[^ ^27^ ^]^ However, to the best of our knowledge, the investigation of RAFT polymerization of fluorinated active ester polymers and especially their PPM has not been studied yet in a continuous flow context. Current research in the field of automated RAFT polymerization is often limited to non‐functional commodity monomers such as acrylates,^[^ ^28^ ^]^ acrylamides,^[^ ^29^ ^]^ isoprene,^[^ ^30^ ^]^ and styrene.^[^ ^31^ ^]^

**Scheme 1:**
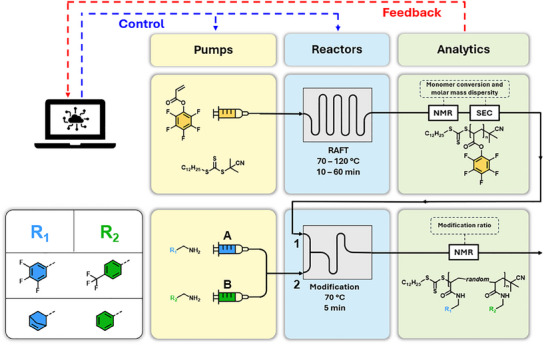
Visualization of the flow reaction setup used in this work. Top: Automated polymerization platform for the RAFT polymerization of pentafluorophenyl acrylate with software‐based feedback and control loop. Bottom: Schematic of the post‐polymerization modification of poly(pentafluorophenyl acrylate) with two different pairs of primary amines. Control over the flow rate facilitates the direct control over the modification ratio of the active polymer.

In this work, we present for the first time the automated self‐optimization of the RAFT polymerization and PPM of PFPA in continuous flow utilizing ^1^H and importantly, ^19^F NMR spectroscopy. The optimum reaction polymerization parameters are recreated and compared to a second independent flow reaction setup, as well as a batch experiment. Second, the obtained active ester‐containing poly(PFPA) precursor are subjected to amidation with two pairs of two different primary amines in a continuous flow reactor. We investigate the precise predictability and control over the modification ratio of poly(PFPA) directly by controlling the flow rate ratios of modifying amine solutions (refer to **Scheme** **1**). Importantly, both ^1^H and ^19^F low field NMR techniques are applicable to determine the modification ratio of poly(PFPA) by identification of characteristic chemical groups in the respective amine. Additionally, obtained materials are isolated and thoroughly characterized with spectroscopic (NMR, IR) and thermal methods (thermogravimetric analysis (TGA), DSC).

## 2 Results and Discussion

### 2.1 Automated Optimization of PFPA Polymerization

**Figure 1:**
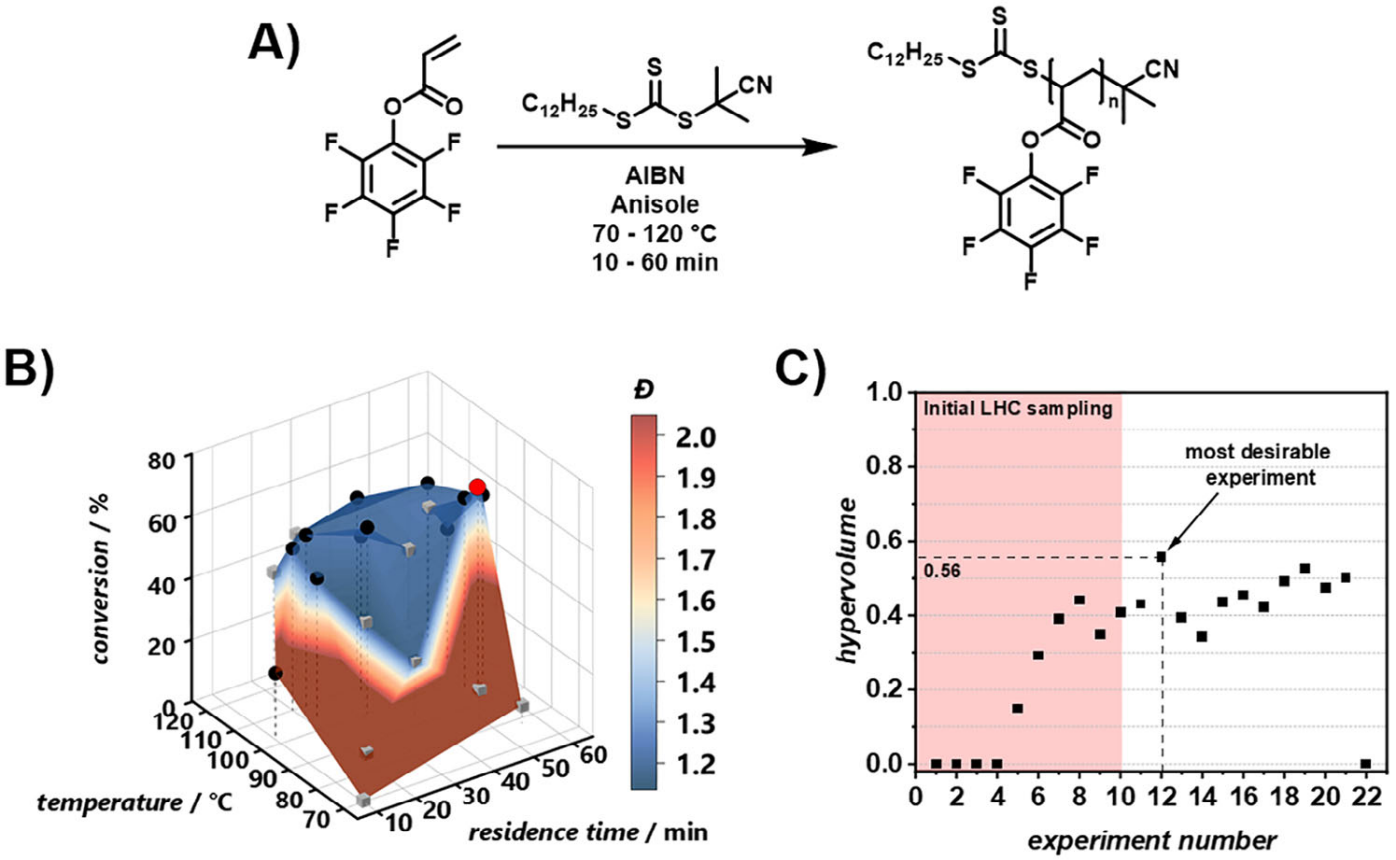
A) Reaction scheme of the polymerization of PFPA with CPDT and AIBN in anisole. B) Monomer conversion and molar mass dispersity for the automated polymerization of PFPA with CPDT and AIBN in anisole. LHC sampling training experiments are shown in grey; optimization results are shown in black. Red: the optimal reaction conditions (parameter space: T = 70 – 120 °C, t_r_ = 10 – 60 min; optimal: T = 94 °C, t_r_ = 56 °C). C) Hypervolume versus experiment number. Highlighted area represents experiments derived from LHC sampling. Experiment 12 (highlighted in red) exhibited the highest hypervolume (0.56), making it the experiment with the most desirable reaction conditions.

Pentafluorophenyl acrylate was synthesized according to literature, and its successful preparation was confirmed using NMR and IR spectroscopy (refer to Figures S3–S6, Supporting Information).^[^ ^32^ ^]^ The synthetic platform designed for the automated optimization of the RAFT polymerization of PFPA featured a stainless steel tubular flow reactor capable of being heated and pressurized. After the reaction, the material stream was analyzed by an in‐line benchtop NMR for monomer conversion and an on‐line SEC for molar mass distribution determination. Flow rates, and consequently residence times (*t*_r_), as well as reaction temperatures, were controlled via a custom‐built MATLAB interface (detailed information can be found in the SI). The RAFT polymerization optimization used a pre‐mixed solution of PFPA, cyanopropyl dodecyl trithiocarbonate (CPDT), and azobisisobutyronitrile (AIBN) in anisole, with concentrations ([PFPA]:[CPDT]: [AIBN]) set at 50:1:0.2 (refer to **Figure** **1**A). The monomer concentration was 20 wt.%. The optimization explored a two‐parameter space: temperatures from 70 to 120 °C and *t*_r_ from 10 to 60 min. NMR spectra were acquired on demand at steady‐state and processed using an automated script for phase and baseline correction.^[^ ^33^ ^]^ Monomer conversion was calculated by comparing the peak integrals in the vinyl region (5.0 – 6.0 ppm) of the stock reaction solution before the reaction with the residual integrals after specific reaction parameters. A programmable switching valve extracted ≈3 µl samples for SEC, with molecular weight data obtained using a rapid‐SEC column equipped with an RI detector. This data, processed automatically, provided the number average molecular weight (*M*_n_), weight average molecular weight (*M*_w_), and *Đ* based on poly(methyl methacrylate) standards. The dataset, generated without human intervention, includes conversion and *Đ* data for temperatures between 70 and 120 °C and *t*_r_ from 10 to 60 min. A color‐mapped surface visualizes the automated screen's search space, highlighting trends in conversion (z‐axis) and *Đ* (color) (refer to Figure 1B). The optimization was carried out with a Thompson sampling efficient multi‐objective optimization (TSEMO) algorithm which uses a Gaussian process‐based model to select future experiments based on initial training data.^[^ ^34^ ^]^ The algorithm's goal is to find reaction parameter sets that resemble the utopian solution to the two‐objective problem (conversion = 100%, *Đ* = 1.0) in the predefined parameter space (T  = 70 – 120 °C, *t*_r_ = 10 – 60 min) by exploring the trade‐off between both objectives, resulting in optimum points, the Pareto front.^[^ ^35^ ^]^ TSEMO is characterized by its efficiency in optimizing multi‐objective problems.^[^ ^34^ ^]^ As a stochastic method, it does not guarantee the identification of all possible optima, but instead offers a route to map the Pareto front. We employ it here in conjunction with an automated platform to rapidly map the trade‐off between dispersity and conversion from which we can select the most desirable conditions to proceed to the next stage of the study with. This self‐driving laboratory approach significantly reduces the time and labor involved in optimizing such a process. Ten initial training parameter sets were generated via Latin hypercube (LHC) sampling to obtain experimental conditions randomly distributed over the parameter space. A workflow diagram of an automated optimization experiment can be found in the Supporting Information (refer to Figure S2, Supporting Information). The platform autonomously executed these ten experiments with independent determination of conversion and molecular weight distribution (refer to Figure 1B, gray datapoints). Based on these training results, the algorithm suggested new reaction parameters, which were then executed and evaluated (refer to Figure 1B, black datapoints). This process was iterated, focusing suggested reaction parameters on high reaction temperatures above 90 °C, which the algorithm found more promising in terms of objective trade‐off (refer to Figure S7, Supporting Information). It was found that the polymerization of PFPA in flow required temperatures and *t*_r_ on the upper end of the investigated parameter space to reach substantial conversions (refer to Figure 1B), which then, in turn, resulted in a good control of the polymerization as documented by low polymer dispersities *Đ* ≈ 1.2 (refer to Figure S8, Supporting Information). The optimization was stopped by the user after no more substantial improvement of the reaction was observed. This was the case after 12 optimization iterations when no more meaningful increase in hypervolume was achieved (refer to Figure S9, Supporting Information). The Pareto front consisted of 7 points of which 5 were found by the optimizing algorithm and all of which can be considered optima; but there are some more desirable points within the front from a chemical and polymer material perspective. To select a single optimum point to carry forward for post‐polymerization modification (PPM), the individual hypervolume contribution of each experiment was calculated (refer to Figure 1C; Figure S10, Supporting Information).^[^ ^36^ ^]^ Iteration 12 (T  =  94 °C, *t*_r_ = 56 min) contributed more hypervolume than any other experiment, found as the second experiment suggested by the algorithm.

To validate the optimization results on one hand, and to showcase the universality and power of automated optimization of polymer synthesis in continuous flow on the other hand, the obtained ideal reaction parameters were employed in comparative experiments in two different laboratories with slightly different flow setups in UK and Germany (refer to SI for detailed information). It is important to emphasize in this case that although based on flow chemistry, the reactors comprised a subtly different configuration. A reaction solution with the same reactant concentrations (([PFPA]: [CPDT]: [AIBN]) = 50:1:0.2; 20 wt.% PFPA in anisole) was purged with nitrogen gas and loaded to a flow reactor setup comprised of a syringe pump attached to a heated 3D‐printed metal flow reactor before sample collection (detailed information can be found in the SI). The monomer conversion was followed via the vinyl region of the stock solution of a 60 MHz ^1^H NMR spectrum. The molecular weight distribution was determined via SEC of the crude polymer solution diluted in dimethylacetamide (DMAc). Additionally, a conventional batch polymerization was carried out under the same conditions. Monomer conversion and *Đ* of the optimization reaction, validation, and batch experiments are displayed in **Table** **1**.

**Table 1:** Monomer conversion and molar mass dispersity of the polymerization of PFPA carried out in two different, independent flow reactor setups and in batch. The most desirable reaction parameters found by the optimization algorithm were T = 94 °C and t_r_ = 56 min.

	Optimization (flow, United Kingdom)	Validation (flow, Germany)	Batch
*monomer conversion* / %	66	64	63
*Đ*	1.16	1.26	1.30

It was found that the conversions of both flow polymerizations are in close agreement, despite the different flow reactor setups used. The high reproducibility of the optimization results substantiates the universality of autonomous polymerization automation, even for functional polymers such as poly(PFPA). The difference in molar mass dispersity (deviation ≈8%) can be explained by the compounding effects of using different flow rates, solvents, columns, calibrations used for SEC, on top of the inherent error that is attributed to SEC measurements.^[^ ^37^ ^]^ However, it can be concluded that the polymerization of PFPA in flow proceed more efficiently when compared to a conventional batch reaction due to improved heat transfer facilitated by the high surface‐to‐volume ratio attributed to flow reactions. While these results indicate a good universality of the obtained optimization data, in‐depth identification of reactor fingerprints and scale models by digital twin reactors represents a promising approach of current research to further advance the usage of PFPA in flow polymerizations techniques based on this work.^[^ ^38^ ^]^ Given the versatile active ester functionality of poly(PFPA) toward various applications, the polymerization of PFPA in continuous flow represents a promising approach toward high‐throughput material development profiting from improved efficiency, reproducibility, and safety provided by continuous flow polymerizations.^[^ ^39^ ^]^

### 2.2 General Considerations of the Post‐Polymerization Modification of Poly(PFPA) in Flow

**Figure 2:**
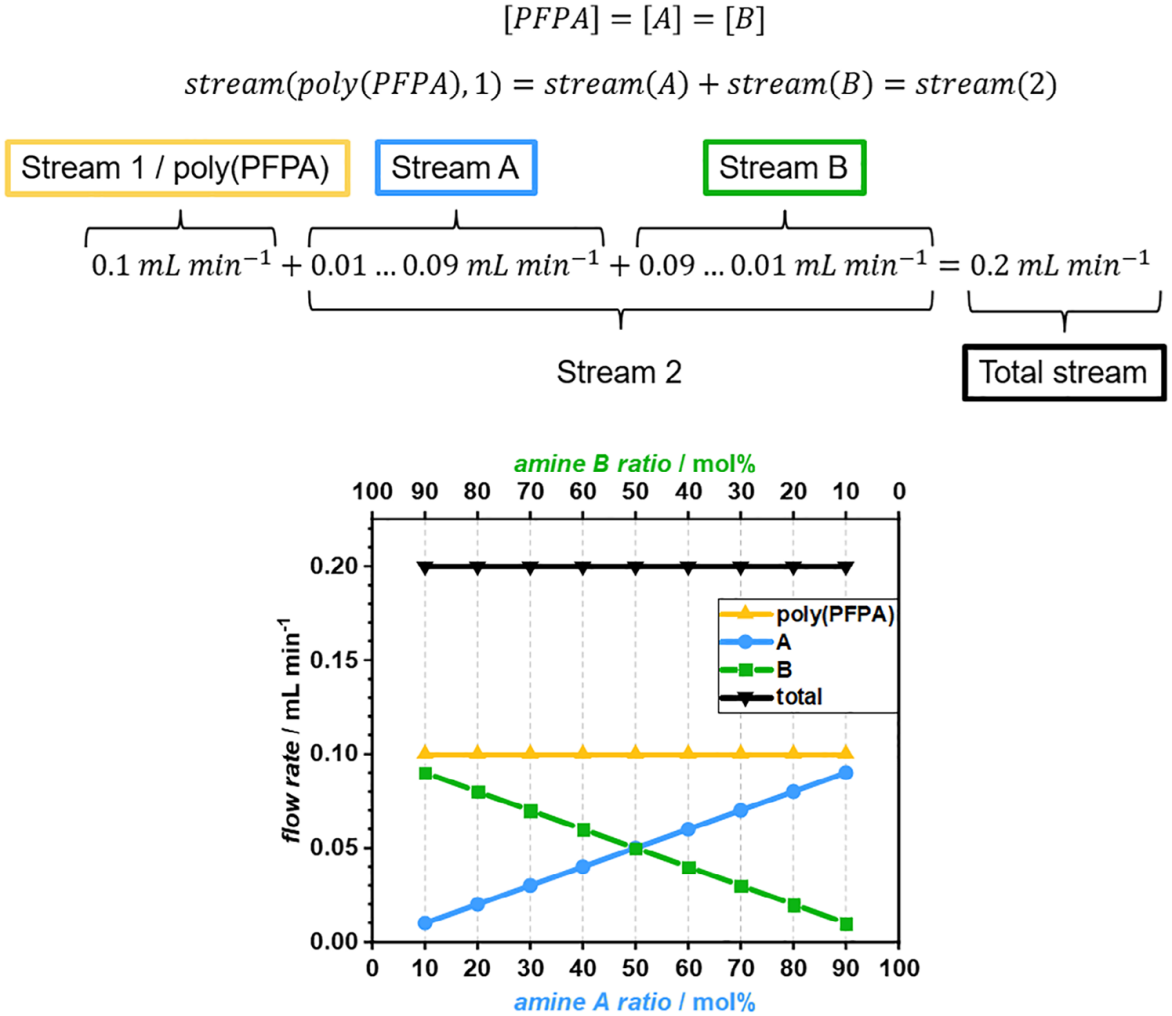
Relations of reactant concentrations and solutions streams for the PPM of poly(PFPA). The sum of streams 1 and 2 always equals 0.2 mL min^−1^ while stream 2 is subdivided into streams A and B which always add up to 0.1 mL min^−1^.

In addition to the polymerization of PFPA, high‐throughput post‐polymerization modification of poly(PFPA) in continuous flow was conducted. A pre‐mixed solution of poly(PFPA) in dimethylformamide (DMF) or DMAc was prepared and loaded to a syringe pump (c = 100 mg mL^−1^, fixed flow rate = 0.1 mL min^−1^). Two additional syringe pumps A and B were loaded with solutions of two different amines in DMF or DMAc, respectively. The amine concentrations in both solutions were chosen to equal the concentration of active ester groups in the polymer solution. In a modification experiment, the stream of syringe A and B were combined before being merged with the polymer solution stream from syringe 1 in a custom 3D‐printed flow channel. The flow rates of streams A and B were always set in a way that their sum equaled the flow rate of stream 1. Stream 2 was divided into ratios of 10 – 90 mol% amine A and 90 – 10 mol% amine B, respectively. Consequently, the resulting total flow rate after mixing was 0.2 mL min^−1^ for every reaction solution composition (refer to **Figure** **2**). The reactor volume of the heated 3D‐printed flow channel was 1.0 mL, resulting in a *t*_r_ of 5 min.

Therefore, the modification of poly(PFPA) with two different amines could be precisely controlled via the flow rates of both amine streams at known concentrations. The lower half of Scheme 1 illustrates the resulting flow path of the reaction setup for the PPM of poly(PFPA) in flow. Importantly, upon direct attachment of the PPM in‐line to the previous polymerization step, a side reaction of the residual PFPA double bonds was observed in the ^1^H NMR spectra after addition of amines. Since acrylates are comprised of a carbonyl‐activated double bond, it is believed that Aza‐Michael addition of the primary amine to the acrylate took place.^[^ ^40^ ^] 19^F NMR spectroscopy revealed the efficient amidation of PFP esters, however his unwanted side reaction falsifies the double bond resonance in the ^1^H NMR and prevented the calculation of the monomer conversion during polymerization (refer to SI, Figure S11, Supporting Information). Therefore, polymerization and PPM were investigated separately. Enlargement of the reaction setup (for example via integration of additional selective analysis steps) or change of the underlying chemical approach would be necessary for a reactor platform that allows analysis of polymerization and PPM in one step.

### 2.3 PPM of Poly(PFPA) Via ^19^F NMR

**Figure 3:**
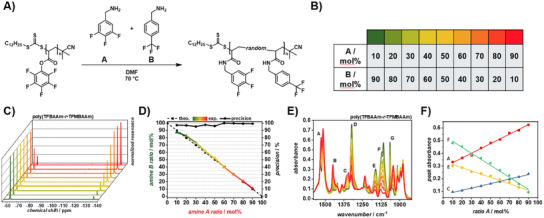
Schematic of the PPM of poly(PFPA) with TFBA and TFMBA. B) Color‐code for the modification of poly(PFPA) with 10 – 90 mol% TFBA and 90 – 10 mol% TFMBA, respectively. C) Normalized ^19^F NMR spectra of poly(TFBAAm‐*r*‐TFMBAAm) in the region between −55 and −140 ppm. The left peaks at ‐61 ppm are attributed to the CF_3_ group of TFMBA while the right peaks at ‐135 ppm are attributed to the *meta*‐fluorine atoms of TFBA. D) Theoretical and experimental modification ratios of poly(PFPA) with TFBA and TFMBA respectively. The black dashed line represents the theoretical input ratios of TFBA and TFMBA which are directly linked to the flow rates of the reactor setup while coloured solid line represents the found ratios in the final polymers. The precision (black circles) shows that almost perfect control over the modification was obtained. E) ATR FT‐IR spectra of poly(TFBAAm‐*r*‐TFMBAAm) in the range of 1580–920 cm^−1^. Characteristic peaks assigned to TFBA and TFMBA, respectively were identified which were found to increase or decrease linearly with the amount of respective amine. F) Linear fits of the peak absorbance against the ratio of TFBA of peaks A, C, E, and F. Plots of peaks B, D, and G were omitted for clarity and can be found in (refer to Figure S16, Supporting Information).

To showcase the versatility and applicability of benchtop ^19^F NMR analysis in continuous flow, an experiment was designed to demonstrate the control over modification of functional poly(PFPA). Based on the considerations detailed above, poly(PFPA) was modified with two different fluorinated primary amines, 3,4,5‐trifluorobenzylamine (TFBA) and 4‐(trifluoromethyl)benzylamine (TFMBA) (refer to **Figure** **3**A). The resulting polymers were termed poly(trifluorobenzyl acrylamide‐*random*‐trifluoromethylbenzyl acrylamide) (poly(TFBAAm‐*r*‐TFMBAAm)). We hypothesized that the difference in chemical environment of aliphatic and aromatic fluorine substituents would allow clear distinction between both amine species. ^19^F NMR spectroscopy of TFBA and TFMBA showed that the aliphatic fluorine atoms of TFMBA were found at a chemical shift of ≈ ‐61 ppm while TFBA showed two resonances at ‐136 and ‐165 ppm caused by *meta* and *para* fluorine atoms, respectively (refer to Figure S12, Supporting Information). By calculation of the integral ratios between TFBA (A) and TFMBA (B) meta‐fluorine atoms therefore allowed the determination of the amine ratio. Given the high reactivity of poly(PFP) against primary amines, we hypothesized that the amidation of PFP active esters could be directly controlled by the flow rates of A and B at known concentrations of PFPA units and amines. Solutions of poly(PFPA), TFBA, and TFMBA were prepared with equal concentrations and loaded to three separate syringe pumps. The flow rates of the three solutions were screened as displayed in Figure 2. The amidation of poly(PFPA) with primary amines is known to happen at room temperature, however as compromise between experiment time and conversion, the PPM of poly(PFPA) was conducted with DMF as solvent at a temperature of 70 °C with a *t*_r_ of 5 min for each reaction solution composition.^[^ ^41^ ^]^ After setting the flow rates of amines A and B to match the desired modification ratio (refer to Figure 2), the reactor was equilibrated with the new solution by passing 4 mL through the whole tubing (20 min). After that, 4 mL of reaction solution were collected in a vial and the polymer samples were isolated by precipitation in cold methanol, centrifuging and drying in vacuum. The modification ratios with TFBA and TFMBA, amine A and B, respectively, were determined via ^19^F NMR spectroscopy. It was found that for every modification reaction, the amidation of PFP active esters was quantitative and no more PFP ester signals were observed in the ^19^F NMR spectra (refer to Figure S13, Supporting Information). The ratios of TFBA to TFMBA in the resulting polymers were calculated by determining the ratio between the two *meta* fluorine atoms from TFBA and the three CF_3_ fluorine atoms from TFMBA (refer to Figure 3B,C; Figure S14, Supporting Information). Importantly, the final ratios of modification in the polymers, very precisely matched the input ratios of TFBA and TFMBA fed into the flow reactor, which were directly linked to the flow rates of the respective amine solutions. The precision with which the modification could be controlled was found to be above 95% throughout the investigated composition range between 10 and 90 mol% (refer to Figure 3D). This excellent precision and control showcased the power of ^19^F NMR in controlling the PPM of poly(PFPA) in flow, which despite its acknowledgement in batch‐based polymer research, has found little application in continuous reaction management. Additionally, ^1^H NMR spectroscopy was employed to investigate its applicability to determine the modification ratios with TFBA and TFBMA, which showed a clear trend of increasing and decreasing resonance associated with the respective aromatic protons (refer to Figure S15, Supporting Information). However, due to the similarity of chemical environment of protons present in TFBA and TFMBA, an isolated integration of the respective protons was not possible. Interestingly, the modification of poly(PFPA) with TFBA and TFMBA at different ratios had an influence on the absorbance of the polymers in the IR region (refer to Figure 3E). It was found that the linear increase of TFBA and the consequential decrease of TFMBA resulted in the linear increase and decrease of respective characteristic bond vibrations. With increasing amount of TFBA, absorbance of C‐C ring vibrations associated with fluorine‐substituted aromatics was found to increase at 1527, 1448, and 1356 cm^−1^, respectively (peaks A to C).^[^ ^42^ ^]^ Meanwhile, the decreasing amount of TFMBA resulted in a decrease of IR absorbance at wavenumbers attributed to CF_3_ groups at 1322, 1162, 1120, and 1065 cm^−1^, respectively (peaks D to G).^[^ ^43^ ^]^ The correlation between peak absorbance and amine ratio was found to be linear, which emphasized the applicability of IR spectroscopy to control the PPM of poly(PFPA) in continuous flow and broadens the range of potential modifying agents by selective analysis of characteristic functional groups incorporated into the parent polymer (refer to Figure 3F; Figure S16, Supporting Information). SEC of poly(TFBAAm‐*r*‐TFMBAAm) showed an overall increase in molecular weight with incorporation of the heavier TFMBA groups, which was in line with the proposed modification of poly(PFPA) while the dispersity did not significantly change (refer to Figures S17, S18, Supporting Information). Thermal properties of modified polymers were investigated using TGA and DSC. TGA revealed a two‐step thermal decomposition of poly(TFBAAm‐*r*‐TFMBAAm) with the first decomposition beginning at ≈ 140 °C (*T*_5%_ ≈ 155 °C). Interestingly, this first decomposition was dependent on the composition of poly(TFBAAm‐r‐TFMBAAm). The higher the content of TFBA, the higher the weight loss of the first thermal decomposition. The second thermal decomposition started at ≈ 340 °C and resulted in the complete decomposition of all polymers (*T*_95%_ ≈ 436 °C) (refer to Figure S19, Supporting Information). DSC analysis revealed a *T*_g_ of 20 °C for all poly(TFBAAm‐*r*‐TFMBAAm) compositions (refer to Figure S20, Supporting Information). Therefore, the thermal properties of poly(TFBAAm‐*r*‐TFMBAAm) could not be significantly tuned, which is believed to be caused by the similarity of TFBA and TFMBA which do not significantly influence chain mobility and thermal stability compared to each other. Nevertheless, the strategy reported herein represents a versatile approach to modifying poly(PFPA) in a highly controlled manner facilitated by ^19^F NMR and IR spectroscopy in combination with continuous flow polymer reaction management.

### 2.4 PPM of Poly(PFPA) Via ^1^H NMR

**Figure 4:**
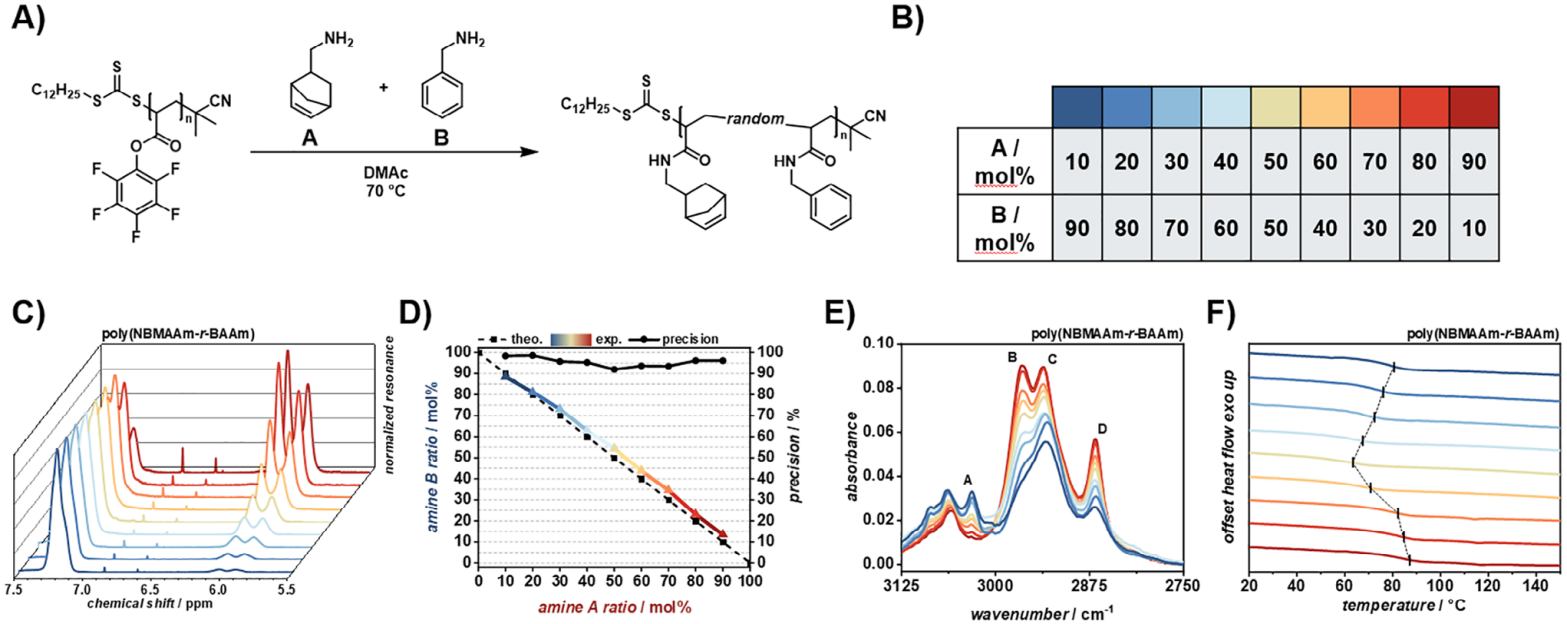
A) Reaction scheme of the PPM of poly(PFPA) with NBMA and BA in DMAc at 70 °C. B) Color‐code for the modification of poly(PFPA) with 10 – 90 mol% NBMA and 90 – 10 mol% BA, respectively. C) Normalized ^1^H NMR spectra of poly(NBMAAm‐*r*‐BAAm) in the region between 7.5 and 5.5 ppm. The left peak at around 7.25 ppm is attributed to the aromatic ring of BA while the right peaks at around 6 ppm are attributed to double bond protons of NBMA. D) Theoretical and experimental modification ratios of poly(PFPA) with NBMA and BA respectively. The black dashed line represents the theoretical input ratios of NBMA and BA which are directly linked to the flow rates of the reactor setup while coloured solid line represents the found ratios in the final polymers. The precision (black circles) shows that almost very good control over the modification was obtained. E) ATR FT‐IR spectra of poly(NBMAAm‐*r*‐BAAm) in the range of 3125–2750 cm^−1^. Peaks assigned to NBMA and BA, respectively were identified which were found to increase or decrease linearly with the amount of respective amine. Linear fits of the ratio of NBMA against the peak absorbance of peaks A – D can be found in the supporting information (refer to Figure S23, Supporting Information). F) DSC thermograms of poly(NBMAAm‐*r*‐BAAm) polymers showing the tunability of *T*_g_ (black line) with the modification ratio of poly(PFPA) with NBMA and BA.

Building on the strategy presented above, we designed an experiment to additionally utilize ^1^H NMR spectroscopy to trace the modification ratio of poly(PFPA). We hypothesized that by employing modifying agents with different distinguishable proton resonances, we would be able to expand the range of usable amines beyond fluorinated compounds. Given the distinct characteristic resonance area of aromatics and olefins, we believe that these two chemical moieties would be distinguishable in ^1^H NMR spectra, therefore allowing quantitative integration and calculation of modification ratios via aromatic and C═C double bond signals. It was found that a mixture of 5‐norbornenyl‐2‐methylamine (NBMA) and benzylamine (BA) exhibits separated aromatic and olefin proton resonances, applicable for integration and ratio calculation (refer to Figure S21, Supporting Information). Consequently, the PPM of poly(PFPA) with NBMA and BA was carried out in continuous flow while screening the ratio of NBMA (A) to BA (B) (refer to **Figure** **4**A,B). The obtained polymers were termed poly(5‐norbornenyl‐2‐methyl)acrylamide‐*random*‐benzyl acrylamide) (poly(NBMAAm‐*r*‐BAAm)). Solutions of poly(PFPA), NBMA, and BA were prepared with equal concentrations and loaded to three separate syringe pumps. The flow rates of the three solutions were screened as displayed in Figure 2. PPM of poly(PFPA) was conducted with DMAc as solvent rather than DMF, to ensure that solvent peaks would not overlap with the resonance area of aromatic protons (δ aromatic protons ≈ 7 – 8 ppm; δ DMF‐CH proton ≈ 8 ppm; DMAc‐*α*‐CH_3_ protons ≈ 2 ppm).^[^ ^44^ ^]^ The reaction temperature was set to 70 °C with a *t*_r_ of 5 min for each reaction solution composition. Collected polymer samples were isolated by precipitation into cold methanol, centrifuging and drying in vacuum. The modification ratios with amine NBMA and BA respectively, were determined via ^1^H NMR spectroscopy. Figure 4C and Figure S22 (Supporting Information) shows the normalized ^1^H NMR spectra of poly(NBMAAm‐*r*‐BAAm) in the area of C═C double bond and aromatic protons were the resonance between 7.00 and 7.50 ppm can be attributed to the five aromatic protons of BAAm while the split signal between 5.75 and 6.25 ppm was assigned to the two double bond protons of the norbornenyl group of NBMAAm. Importantly, the final ratios of modification in the polymers, very well matched the input ratios of NBMA and BA fed into the flow reactor. However, the modification could be controlled but was found to be slightly less precise than compared to the PPM of poly(PFPA) with fluorinated amines. The amount of incorporated BA was found to be slightly higher than would be dictated by the input ratio (refer to Figure 4D). This can be explained by the slightly different nucleophilicity of amines with aromatic and aliphatic neighboring groups.^[^ ^45^ ^]^ Also, the loss of C═C double bond protons due to side reactions of the norbornene moieties cannot be ruled out completely, which however is believed to be negligible since norbornene reactions usually require harsh conditions or presence of catalysts.^[^ ^46^ ^]^ Nevertheless, the precision with which the modification of poly(PFPA) with NBMA and BA could be controlled was found to be above 90% throughout the investigation range of 10 – 90 mol% NBMA or 90 – 10 mol% BA, respectively. IR analysis of poly(NBMAAm‐*r*‐BAAm) polymers was also conducted in order to investigate the chance in absorbance with varying modification ratios of poly(PFPA) (refer to Figure 4E). C‐H stretch vibrations assigned to C═C double bonds at 3030 cm^−1^ (peak A) were found to decrease with decreasing amount of aromatic benzyl groups in the polymers.^[^ ^47^ ^]^ This finding was in line with the proposed modification of poly(PFPA) since C═C double bonds were more pronounced in BA compared to NBMA. Peaks C to D at 2965, 2936, and 2868 cm^−1^ could be assigned to the asymmetric CH_2_ stretch, ring CH stretch, and symmetric CH_2_ stretch vibrations of norbornene rings, respectively, which increased with increasing input ratio of NBMA.^[^ ^48^ ^]^ The correlation between peak absorbance and amine ratio was found to be linear (refer to Figure S23, Supporting Information) which substantiates the linear modification ratio proclaimed by the experimental design. However, it needs to be noted that due to the structural similarity of NBMA and BA, IR analysis of poly(NBMAAm‐*r*‐BAAm) remains challenging. Next, SEC analysis of poly(NBMAAm‐*r*‐BAAm) revealed a slight overall increase in molecular weight with increasing amount of NBMA due to the incorporation of the heavier norbornene groups, consistent with the expected modifications of poly(PFPA). The dispersity, however, remained largely unchanged (refer to Figures S24, S25, Supporting Information). Further, the thermal decomposition profiles of poly(NBMAAm‐*r*‐BAAm) polymers were investigated by TGA. Interestingly, an increase in norbornene moieties in the polymers led to an enhancement of the expression of gradual thermal decomposition with *T*_5%_ values ranging from 107 °C to 161 °C (detailed information can be found in Figure S26; Table S3, Supporting Information). The multistep decomposition of poly(NBMAAm‐*r*‐BAAm) with higher NBMAAm contents (above 50 mol%) is believed to be caused by the thermal lability of norbornene moieties due to ring strain compared to aromatic benzyl groups.^[^ ^49^ ^]^ Phase transition behavior of poly(NBMAAm‐*r*‐BAAm) polymers was investigated by DSC. Interestingly, the *T*_g_ of poly(NBMAAm‐*r*‐BAAm) was found to be dependent on the ratio between NBMAAm and BAAm in the final polymer (refer to Figure 4F). Increasing the amount of NBMAAm from 10 to 50 mol% led to a gradual decrease of *T*_g_ from 80 to 63 °C. Upon further increase of NBMAAm from 50 to 90 mol%, the *T*_g_ was found to increase back from 63 to 87 °C, resulting in a V‐shaped *T_g_*‐profile over amine ratio (refer to Figure S27, Supporting Information). We hypothesize that the change in *T*_g_ is caused by a change in chain mobility due to side group effects. Increasing the ratio of foreign pendant chain structures may lead to a hinderance of optimal chain orientation which causes less stable chain stacking, increased chain mobility and therefore leads to a minimum *T*_g_ at a 50 mol% modification mixture.^[^ ^50^ ^]^ Given the high precision of control over the modification of poly(PFPA) demonstrated in this work, the tunability of polymer materials properties facilitated by continuous flow modification represents a promising approach toward novel high‐throughput materials synthesis and analysis.

## 3 Conclusion

An automated flow reactor equipped with orthogonal online analytics was successfully used for machine‐learning directed self‐optimization of the polymerization of PFPA. Subsequent implementation of the algorithmically elucidated optimal polymerization parameters with a configurationally different automated flow‐reactor in another lab yielded comparable materials. Moreover, whilst equivalent batch experiments achieved a comparable conversion, there was an associated undesirable broadening of molar mass dispersity. Additionally, an adapted flow platform allowed precise control, with over 90% accuracy, over the PPM of poly(PFPA) via amidation of with primary amines. In this case, ^19^F and ^1^H NMR spectroscopy was employed to validate the applicability of presented strategy for poly(PFPA) modification using both fluorinated and non‐fluorinated modifying agents bearing characteristic NMR‐active groups. IR spectroscopy of the modified polymers revealed linear absorbance band intensities of characteristic bond vibrations with linear degree of modification with the respective functional groups, substantiating the power of IR spectroscopy in flow polymer chemistry. Ultimately, thermal decomposition and phase transitions of modified polymers were investigated by TGA and DSC, respectively, which proved tunability of material properties by the controlled modification of poly(PFPA) in flow. While this work is limited to the amidation of poly(PFPA) homopolymers with specified amine pairs, we are convinced that the strategy presented herein can be applied to other active ester containing polymers such as NHS‐based systems and with more complex mixtures of amines tracible by NMR spectroscopy. Also the extension to transesterification or thiolysis may be possible going forward which may allow construction of highly complex architectures derived from active ester‐containing polymers by virtue of flow chemistry. Overall, this work demonstrates an exciting approach toward high‐throughput investigation of novel functional and tailored polymeric materials. Importantly, it also exemplifies opportunities for more sustainable and efficient collaborative research between global labs with complementary expertise.

## Conflict of Interest

The authors declare no conflict of interest.

## Supporting information



Supporting Information

## Data Availability

The data that support the findings of this study are openly available in RADAR4Chem at https://doi.org/10.22000/s2myp3gw533d4vq0, reference number 51.

## References

[1] a) C. W. Coley , D. A. Thomas , J. A. M. Lummiss , J. N. Jaworski , C. P. Breen , V. Schultz , T. Hart , J. S. Fishman , L. Rogers , H. Gao , R. W. Hicklin , P. P. Plehiers , J. Byington , J. S. Piotti , W. H. Green , A. John Hart , T. F. Jamison , K. F. Jensen , Science 2019, 365; 10.1126/science.aax1566 31395756

[2] R. F. Service , Science 2023, 382, 987.38033067

[3] L. Zhichao , M. Dong , L. Xiongjun , Z. Lu , Commun. Mater. 2024, 5, 76.

[4] D. Paul , G. Sanap , S. Shenoy , D. Kalyane , K. Kalia , R. K. Tekade , Drug Discov. Today 2021, 26, 80. 33099022 10.1016/j.drudis.2020.10.010 PMC7577280

[5] a) M. Fichtner , K. Edström , E. Ayerbe , M. Berecibar , A. Bhowmik , I. E. Castelli , S. Clark , R. Dominko , M. Erakca , A. A. Franco , A. Grimaud , B. Horstmann , A. Latz , H. Lorrmann , M. Meeus , R. Narayan , F. Pammer , J. Ruhland , H. Stein , T. Vegge , M. Weil , Adv. Energy Mater. 2022, 12, 2102904;

[6] a) A. F. de Almeida , R. Moreira , T. Rodrigues , Nat. Rev. Chem. 2019, 3, 589;

[7] D. Cambié , C. Bottecchia , N. J. W. Straathof , V. Hessel , T. Noël , Chem. Rev. 2016, 116, 10276. 26935706 10.1021/acs.chemrev.5b00707

[8] a) M. B. Plutschack , B. Pieber , K. Gilmore , P. H. Seeberger , Chem. Rev. 2017, 117, 11796; 28570059 10.1021/acs.chemrev.7b00183

[9] F. Parveen , N. Watson , A. M. Scholes , A. G. Slater , Curr. Opin. Green Sustain. Chem. 2024, 48, 100935.

[10] L. Yu , B. Chen , Z. Li , Y. Su , X. Jiang , Z. Han , Y. Zhou , D. Yan , X. Zhu , R. Dong , Giant 2024, 18, 100252.

[11] S. Perrier , Macromolecules 2017, 50, 7433.

[12] Y.‐N. Zhou , J.‐J. Li , Y.‐Y. Wu , Z.‐H. Luo , Chem. Rev. 2020, 120, 2950. 32083844 10.1021/acs.chemrev.9b00744

[13] M. Hartlieb , Macromol. Rapid Commun. 2022, 43, 2100514. 10.1002/marc.202100514 34750911

[14] a) Y. Wang , M. Fantin , S. Park , E. Gottlieb , L. Fu , K. Matyjaszewski , Macromolecules 2017, 50, 7872; 29977098 10.1021/acs.macromol.7b02005 PMC6028042

[15] a) H. Mohapatra , M. Kleiman , A. P. Esser‐Kahn , Nat. Chem. 2017, 9, 135;

[16] a) G. Delaittre , C. Dire , J. Rieger , J.‐L. Putaux , B. Charleux , Chem. Commun. 2009, 10.1039/B903040A; 19436899

[17] J. Xu , C. Fu , S. Shanmugam , C. J. Hawker , G. Moad , C. Boyer , Angew. Chem., Int. Ed. 2017, 56, 8376. 10.1002/anie.201610223 27925363

[18] a) M. Rubens , J. H. Vrijsen , J. Laun , T. Junkers , Angew. Chem. Int. Ed. 2019, 58, 3183; 10.1002/anie.201810384 30375134

[19] J. F. R. van Guyse , Y. Bernhard , A. Podevyn , R. Hoogenboom , Angew. Chem., Int. Ed. 2023, 62, 202303841. 10.1002/anie.202303841 37335931

[20] a) E. Blasco , M. B. Sims , A. S. Goldmann , B. S. Sumerlin , C. Barner‐Kowollik , Macromolecules 2017, 50, 5215;

[21] A. Das , P. Theato , Macromolecules 2015, 48, 8695.

[22] (Eds: H.‐A. Klok , P. Theato ), Functional Polymers by Post‐Polymerization Modification. Concepts, Guidelines, and Applications, Wiley‐VCH, Weinheim, 2013.

[23] K. Kuroda , M. Ouchi , Angew. Chem. Int. Ed. 2024, 63, 202316875. 10.1002/anie.202316875 37971837

[24] Q. Zhuo , J. Yang , X. Zhou , T. Shima , Y. Luo , Z. Hou , J. Am. Chem. Soc. 2023, 145, 22803. 37797654 10.1021/jacs.3c08715

[25] Y. Ling , J. Liu , Y.u Dong , Y. Chen , J. Chen , X. Yu , B. Liang , X. Zhang , W. An , D. Wang , S. Feng , W. Huang , Adv. Mater. 2023, 35, 2303641. 10.1002/adma.202303641 37347620

[26] M. Reis , F. Gusev , N. G. Taylor , S. H. Chung , M. D. Verber , Y. Z. Lee , O. Isayev , F. A. Leibfarth , J. Am. Chem. Soc. 2021, 143, 17677. 34637304 10.1021/jacs.1c08181 PMC10833148

[27] A. I. Silva Terra , M. Rossetto , C. L. Dickson , G. Peat , D. Uhrín , M. E. Halse , ACS Meas. Sci. Au 2023, 3, 73. 36817010 10.1021/acsmeasuresciau.2c00055 PMC9936801

[28] a) J. H. Vrijsen , I. A. Thomlinson , M. E. Levere , C. L. Lyall , M. G. Davidson , U. Hintermair , T. Junkers , Polym. Chem. 2020, 11, 3546;

[29] a) S. Parkinson , S. T. Knox , R. A. Bourne , N. J. Warren , Polym. Chem. 2020, 11, 3465;

[30] F. Lauterbach , M. Rubens , V. Abetz , T. Junkers , Angew. Chem. Int. Ed. 2018, 57, 14260. 10.1002/anie.201809759 30168247

[31] A.‐L. Buckinx , M. Rubens , N. R. Cameron , C. Bakkali‐Hassani , A. Sokolova , T. Junkers , Polym. Chem. 2022, 13, 3444.

[32] F. D. Jochum , P. Theato , Macromolecules 2009, 42, 5941.

[33] S. T. Knox , S. Parkinson , R. Stone , N. J. Warren , Polym. Chem. 2019, 10, 4774.

[34] A. D. Clayton , A. M. Schweidtmann , G. Clemens , J. A. Manson , C. J. Taylor , C. G. Niño , T. W. Chamberlain , N. Kapur , A. J. Blacker , A. A. Lapkin , R. A. Bourne , Chem. Eng. J. 2020, 384, 123340.

[35] S. T. Knox , S. J. Parkinson , C. Y. P. Wilding , R. A. Bourne , N. J. Warren , Polym. Chem. 2022, 13, 1576.

[36] Y. Cao , B. J. Smucker , T. J. Robinson , J. Stat. Plan. Inference 2015, 160, 60.

[37] D. D. Bly , H. J. Stoklosa , J. J. Kirkland , W. W. Yau , Anal. Chem. 1975, 47, 1810.

[38] a) Y. Qamsane , J. Moyne , M. Toothman , I. Kovalenko , E. C. Balta , J. Faris , D. M. Tilbury , K. Barton , IEEE Access 2021, 9, 44247;

[39] M. H. Reis , F. A. Leibfarth , L. M. Pitet , ACS Macro Lett. 2020, 9, 123. 35638663 10.1021/acsmacrolett.9b00933

[40] J. Clayden , N. Greeves , S. G. Warren , Organic Chemistry, Oxford University Press, Oxford, New York, Auckland, 2012.

[41] A. Das , P. Theato , Chem. Rev. 2016, 116, 1434. 26305991 10.1021/acs.chemrev.5b00291

[42] P. Brown , E. F. Mooney , Spectrochim. Acta A Mol. Spectrosc. 1968, 24, 1317.

[43] a) D. H. Lemmon , J. Mol. Struct. 1978, 49, 71;

[44] G. R. Fulmer , A. J. M. Miller , N. H. Sherden , H. E. Gottlieb , A. Nudelman , B. M. Stoltz , J. E. Bercaw , K. I. Goldberg , Organometallics 2010, 29, 2176.

[45] F. Brotzel , Y. C. Chu , H. Mayr , J. Org. Chem. 2007, 72, 3679. 17411095 10.1021/jo062586z

[46] V. R. Flid , M. L. Gringolts , R. S. Shamsiev , E. S. Finkelshtein , Russ. Chem. Rev. 2018, 87, 1169.

[47] H. G. Brittain , Cryst. Growth Des. 2009, 9, 3497.

[48] S. F. Parker , K. P. J. Williams , D. Steele , H. Herman , Phys. Chem. Chem. Phys. 2003, 5, 1508.

[49] O. Herbinet , B. Sirjean , F. Battin‐Leclerc , R. Fournet , P.‐M. Marquaire , Energy Fuels 2007, 21, 1406.

[50] a) M. Hirooka , T. Kato , J. Polym. Sci. B Polym. Lett. Ed. 1974, 12, 31;

[51] A. P. Grimm , A Versatile Flow Reactor Platform for Machine Learning Guided RAFT Synthesis and Amidation of Poly(pentafluorophenyl acrylate), Patrick Théato 2024, 10.22000/s2myp3gw533d4vq0. PMC12687679 40198808

