# Supplementary material for: A Versatile Flow Reactor Platform for Machine Learning Guided RAFT Synthesis, Amidation of Poly(Pentafluorophenyl Acrylate)
Source: Macromol Rapid Commun. 2025 Apr 8;46(23):2500264. doi: 10.1002/marc.202500264 (PMC12687679; doi:10.1002/marc.202500264)
Supplement: Supplementary file 1 — Supporting Information [file MARC-46-2500264-s001.docx]

Supporting Information
©Wiley-VCH 2021
69451 Weinheim, Germany

**A Versatile Flow Reactor Platform for Machine Learning Guided RAFT Synthesis and Amidation of Poly(pentafluorophenyl acrylate)**

Alexander P. Grimm, Stephen T. Knox, Clarissa Y. P. Wilding, Harry A. Jones, Björn Schmidt, Olga Piskljonow, Dominik Voll, Christian W. Schmitt, Nicholas J. Warren,* and Patrick Théato*

**Abstract:** Data-driven polymer research has experienced a dramatic upswing in recent years owing to the emergence of artificial intelligence alongside automated laboratory synthesis. However, the chemical complexity of polymers employed in automated synthesis still lacks in terms of defined functionality to meet the need of next generation high performance polymer materials. In this work, we present the automated self-optimization of the reversible addition-fragmentation chain-transfer polymerization of pentafluorophenyl acrylate (PFPA), a versatile polymer building-block enabling efficient post-polymerization modifications. The polymerization system consisted of a computer-operated flow reactor with orthogonal analytics comprising an inline benchtop nuclear magnetic resonance spectrometer, and an online size exclusion chromatography. This setup enabled the automatic determination of optimal polymerization conditions by implementation of a multi-objective Bayesian self-optimization algorithm. The obtained poly(PFPA) was precisely modified by amidation taking advantage of the active PFP ester. By controlling the feed ratios of solutions containing different amines, their incorporation ratio into the polymer, and therefore its resulting properties, could be tuned and predicted, which was shown using NMR, differential scanning calorimetry, and infrared analysis. The described strategy represents a versatile method to synthesize and modify reactive polymers in continuous flow, expanding the range of functional polymer materials accessible by continuous, high-throughput synthesis.

DOI:

Table of Contents

[Table of Contents 2](#_Toc189564361)

[Chemicals 3](#_Toc189564362)

[Experimental procedures 3](#_Toc189564363)

[Synthesis of pentafluorophenyl acrylate (PFPA) 3](#_Toc189564364)

[Automated platform (Leeds, UK) 3](#_Toc189564365)

[Flow polymerization setup (Karlsruhe, Germany) 3](#_Toc189564366)

[Automated polymerization of PFPA (Leeds, UK) 4](#_Toc189564367)

[Flow polymerization of PFPA (Karlsruhe, Germany) 6](#_Toc189564368)

[Post-polymerization modification of poly(PFPA) in flow 7](#_Toc189564369)

[Characterization methods 8](#_Toc189564370)

[NMR 8](#_Toc189564371)

[ATR FT-IR 8](#_Toc189564372)

[TGA 8](#_Toc189564373)

[SEC 8](#_Toc189564374)

[DSC 8](#_Toc189564375)

[Results and Discussion 9](#_Toc189564376)

[Raw data 22](#_Toc189564377)

[Author Contributions 22](#_Toc189564378)

Chemicals

2-Cyano-2-propyl dodecyl trithiocarbonate (BLD Pharmatech, 97 %), 3,4,5-trifluorobenzylamine (BLD Pharmatech, 98 %), 4-(trifluormethyl)benzylamine (BLD Pharmatech, 97 %), 5-norbornenyl-2-methylamine (mixture of isomers) (TCI, ≥ 98 %), acryloyl chloride (Sigma-Aldrich, ≥ 97 %), anisole (Sigma-Aldrich, anhydrous, ≥ 99.7 %), benzylamine (Sigma-Aldrich, 99 %), dichloromethane (VWR, 99 %), dimethylacetamide (Carl Roth, 99 %), dimethylformamide (Acros Organics, anhydrous, 99.5 %), dimethylsulfoxide-d6 (Eurisotop, 99.8 %), magnesium(II) sulfate (Carl Roth, 99 %), methanol (VWR, 98 %), pentafluorophenol (BLD Pharmatech, 98 %), petrol ether (VWR, 98 %), triethylamine (Sigma-Aldrich, 99 %) were used as received. Azobisisobutyronitrile (Sigma-Aldrich, 98 %) was recrystallized before use. Pentafluorophenyl acrylate was synthesized according to literature.^[1]^

Experimental procedures

Synthesis of pentafluorophenyl acrylate (PFPA)

In a round bottom flask equipped with a magnetic stirring bar, pentafluorophenol (30 g, 0.16 mol, 1.00 eq) was dissolved in DCM (280 mL). The mixture was cooled to 0 °C and triethylamine  (24 mL, 0.17 mol, 1.10 eq) was added slowly. Acryloyl chloride (15 mL, 0.17 mol, 1.10 eq) was added dropwise via a dropping funnel, and the mixture was stirred overnight to reach ambient temperature. The reaction mixture was washed with water (3 x 300 mL), dried over MgSO_4_, filtered, and concentrated under reduced pressure. The product was obtained after column chromatography with petrol ether as eluent (80 %).

^1^H NMR (400 MHz, CDCl_3_): δ (ppm) = 6.72 (dd, 1H), 6.37 (m, 1H), 6.18 (dd, 1H).

^13^C NMR (100 MHz, CDCl_3_): δ (ppm) = 161.83, 142.68, 140.95, 140.17, 139.33, 138.44, 136.80, 135.63, 125, 50.

^19^F NMR (376 MHz, CDCl_3_) δ (ppm) = -152.93 (m, 2F, *ortho*-F), -158.41 (t, 1F, *para*-F), -162.77 (m, 2F, *meta*-F).

ATR FT-IR: ν (cm^-1^) = 1770 *w br*, 1657 *vw*, 1634 *vw*, 1515 *vs*, 1471 *vw*, 1406 *w*, 1293 *vw br*, 1218 *w br*, 1149 *vw*, 1111 *m br*, 1069 *vw*, 1030 *w*, 992 *vs br*, 870 *vw br*, 797 *vw*, 732 *vw*, 636 *vw br*, 618 *vw*.

Automated platform (Leeds, UK)

The reactor platform used for the optimization of the polymerization of PFPA has previously been described elsewhere.^[2,3]^ Generally, the automated reactor platform included one JASCO-PU 980 pump, and an aluminum heating block equipped with two Elmatic Max K cartridge heaters regulated by a Eurotherm 3210 temperature controller. The reactor vessel was formed by coiling 1/16” stainless steel tubing with an internal volume of 1 mL around the heating block. The equipment was managed by a custom MATLAB interface, enabling autonomous kinetic experiments. GPC data was collected online using an Agilent Rapide M with a 5 µm guard column employing RI detection by a Knauer K2301 RI detector. The eluent used was DMF treated with 0.1 w/w % LiBr. The GPC system was calibrated using poly(methyl methacrylate) standards. Conversion monitoring was conducted with a Magritek SpinSolve Ultra 60 (^1^H: 7 µs excitation pulse, 6.4 s acquisition time, and 10 s repetition time for 2 scans). Kinetic samples were collected after three reactor volumes to ensure steady-state conditions.

Flow polymerization setup (Karlsruhe, Germany)

The setup located at Karlsruhe Institute of Technology (Germany) was comprised of three CETONI Nemesys M syringe pumps (volumes = 50, 25, 25 mL) equipped with a 3-way high-pressure Contiflow ball valve from CETONI GmbH (Korbussen, Germany). The 50 mL syringe pump was attached to a 3D-printed metal flow reactor made from 316L stainless steel (Type 1.4404) and fabricated by selective laser sintering (SLS) (refer to Figure S1). The reactor volume was 1 mL with a flow channel radius of 1.00 mm. The reactor was heated by custom-made heating jackets made from aluminum equipped with 4 heating cartridges (160 W heating power each) with integrated Type K thermocouples from Horst GmbH (Lorsch, Germany). Temperature control was done by a F4T 4-zone benchtop temperature controller from Zesta Engineering Ltd. (Mississauga, ON, USA). The flow reactor was placed inside the heating jackets and screwed together firmly for good contact and heat transfer. The output streams of the two 25 mL syringe pumps were fed together with a Swagelok T-piece which was attached to a 3D-printed mixing channel made from a commercial Formlabs Form 2 SLA 3D-printer (Berlin, Germany) with Clear V4 resin.


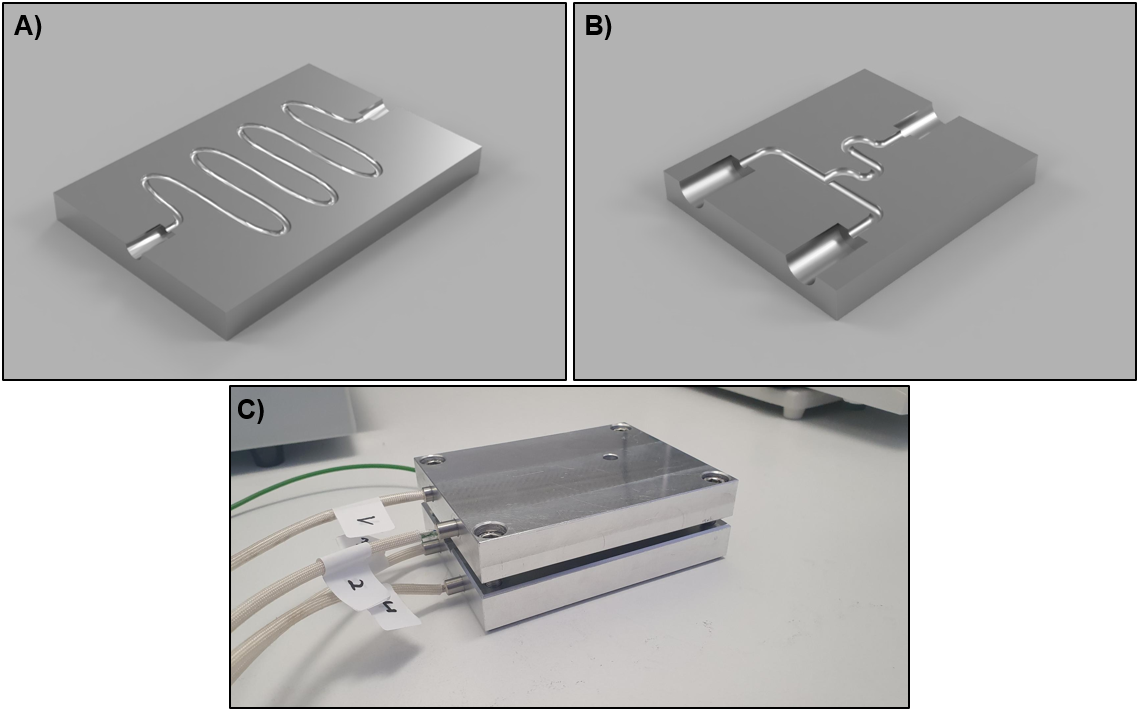


**Figure S1.** 3D-rendered cross-sections of A) the reactor used for heated flow-reactions (material: 316L, 1.4404 stainless steel, channel radius: 1 mm) and B) the mixer used to combine streams at ambient temperature (material: commercial 3D-printed methacrylic plastics, channel radius: 1 mm). C) Photography of the heating jackets (material: aluminium) with 4 inserted heating cartridges.

Automated polymerization of PFPA (Leeds, UK)

The automated polymerization was carried out in the laboratories of the University of Leeds (United Kingdom). In a 500 mL round bottom flask, PFPA (58.20 g, 0.24 mol, 50.0 eq), CPDT (1.71 g, 4.93 mmol, 1.00 eq), and AIBN (181 mg, 1.10 mmol, 0.02 eq) were dissolved in 232 mL anhydrous anisole. The reaction mixture was degassed by purging with nitrogen gas for 15 minutes. The flask containing the reaction mixture was kept under nitrogen gas and connected to the flow platform by submerging one end of a steel tubing which was connected to one of the pumps. The complete tubing of the reactor platform was purged with the reaction solution and initial ^1^H spectra were recorded from which the monomer conversion was calculated. The parameter space was set to a reaction time of 10 – 60 minutes (resolution = 2 minutes) and a reaction temperature of 70 – 120 °C (resolution = 2 °C). The number of initial training steps was set to 10 for Latin hypercube sampling. The process of the autonomous optimization is visualized below in the form of a flow diagram. Each analysis was conducted at identical conditions with the NMR performed on the production stream flowing at 0.1 mL min^-1^. Good quality spectra were obtained on a slow flowing product stream by using 1/8’’ PFA tubing to avoid issues that can arise when using flow cells with more complicated geometries.^[2,4]^ The SEC setup used a rapid column and as such a single injection took 6 mins for a result. To improve reproducibility of measurement, a triple overlaid injection sequence was used which allowed for triplicate analysis in 12 mins. The entire analysis window required 15 mins and as such consumed ~1.5 mL of material. In total, each datapoint required < 9 mL of reaction solution. The workflow of the automated optimization is visualized in Figure S2.


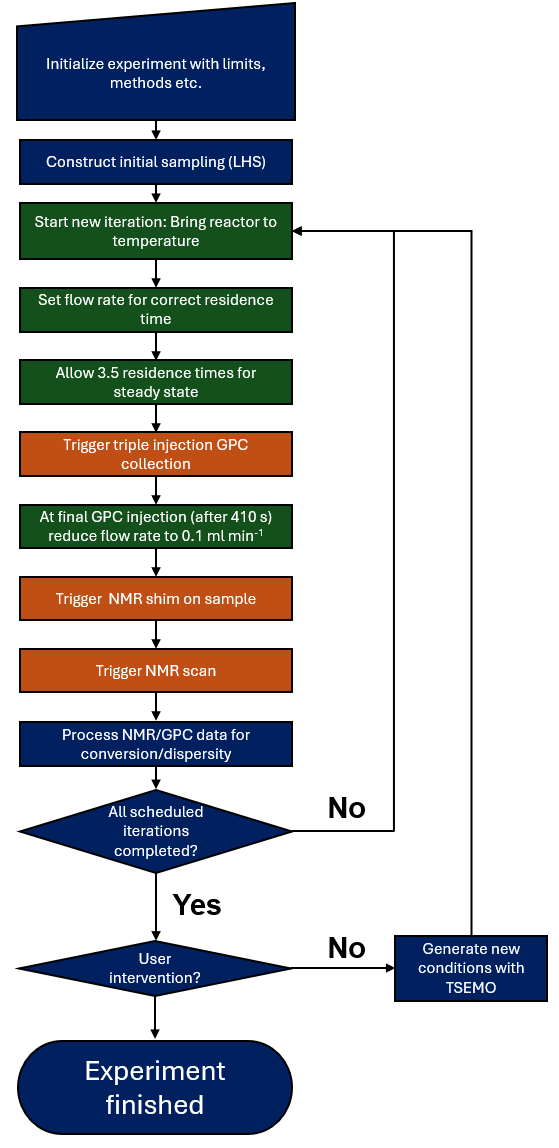


**Figure S2.** Workflow visualization of an automated optimization experiment carried-out at the University of Leeds. Key steps or the process are laid out. Blue: computational processes; green: reactor processes; orange: analytical processes. The analytical element of the loop takes approximately 15 minutes, consuming ~1.5 mL of reaction solution.

**Table S1.** Monomer conversion, number average molecular weight, dispersity, and hypervolume of the automation results from the polymerization of PFPA. The first ten experiments (highlighted in gray) were the training experiments, chosen by Latin hypercube sampling. Experiment number twelve (red) was found to be the most desirable experiment.

| *Experiment number* | *temperature* / °C | *residence time* / min | *Monomer conversion* / % | *M*_n_ / g mol^-1^ | *Đ* | Hypervolume |
| --- | --- | --- | --- | --- | --- | --- |
| 1 | 70 | 10 | 0.6 | 0 | 2 | 0 |
| 2 | 78 | 56 | 5.9 | 0 | 2 | 0 |
| 3 | 82 | 48 | 12.0 | 0 | 2 | 0 |
| 4 | 86 | 22 | 0.5 | 0 | 2 | 0 |
| 5 | 94 | 40 | 17.1 | 3690 | 1.12 | 0.149 |
| 6 | 98 | 30 | 31.9 | 3570 | 1.09 | 0.292 |
| 7 | 102 | 44 | 46.3 | 4600 | 1.15 | 0.391 |
| 8 | 106 | 52 | 53.6 | 4740 | 1.18 | 0.441 |
| 9 | 114 | 18 | 42.9 | 4130 | 1.19 | 0.347 |
| 10 | 120 | 28 | 46.9 | 4020 | 1.13 | 0.407 |
| 11 | 118 | 60 | 50.4 | 4320 | 1.15 | 0.430 |
| 12 | 94 | 56 | 66.4 | 6230 | 1.16 | 0.557 |
| 13 | 104 | 22 | 45.4 | 4450 | 1.13 | 0.395 |
| 14 | 108 | 58 | 43.0 | 5000 | 1.20 | 0.343 |
| 15 | 110 | 20 | 52.1 | 4610 | 1.16 | 0.436 |
| 16 | 118 | 42 | 53.7 | 4470 | 1.15 | 0.455 |
| 17 | 108 | 36 | 50.4 | 5050 | 1.16 | 0.422 |
| 18 | 100 | 32 | 59.9 | 5760 | 1.18 | 0.491 |
| 19 | 96 | 54 | 62.2 | 6180 | 1.16 | 0.525 |
| 20 | 108 | 22 | 56.9 | 5140 | 1.17 | 0.473 |
| 21 | 98 | 60 | 59.3 | 5820 | 1.16 | 0.500 |
| 22 | 102 | 10 | 21.4 | 4300 | 2 | 0 |

Flow polymerization of PFPA (Karlsruhe, Germany)

The optimal polymerization parameters obtained from the automated optimization of the polymerization of PFPA were employed in a second, independent flow reactor setup allocated at Karlsruhe Institute of Technology (Germany). In a 25 mL round bottom flask, PFPA (6.27 g, 26.33 mmol, 50.0 eq), CPDT (184 mg, 0.531 mmol, 1.00 eq), and AIBN (19.5 mg, 0.118 mmol, 0.02 eq) were dissolved in 25 mL anhydrous anisole. The reaction mixture was degassed by purging with nitrogen gas for 15 minutes. A sample was withdrawn for measuring a pilot 60 MHz ^1^H NMR spectrum for monomer conversion calculation before loading to the 50 mL syringe pump of the flow setup. The reactor was heated to 94 °C and the flowrate was set to 0.01786 mL min^-1^ which resembles a residence time of 56 minutes. Three reactor volumes were passed through the reactor to ensure steady-state and around 1 mL of reaction mixture was collected for calculation of monomer conversion and SEC analysis.

Post-polymerization modification of poly(PFPA) in flow

2.00 g poly(PFPA) were dissolved in 20 mL anhydrous DMF and loaded to syringe pump 1. 1.02 g 3,4,5-Trifluorobenzylamine (6.30 mmol) and 1.10 g 4-(Trifluoromethyl)benzylamine (6.30 mmol) were separately dissolved in 15 mL anhydrous DMF and the solutions were loaded to syringe pumps 2 and 3, respectively. The 3D-printed metal flow reactor was heated to 70 °C and dosing of the syringe pumps was started (flow rates see below, Table S2). After each flow rate change, a total of 4 mL was passed through the system before 4 mL of sample were collected. The polymers were isolated by precipitation in cold methanol, followed by centrifugation and drying in vacuum at 40 °C for 18 hours.

PPM of poly(PFPA) (2.00 g in 20 mL) with norbornenylmethylamine (776 mg, 6.30 mmol in 15 mL) and benzylamine (675 mg, 6.30 mmol in 15 mL) were carried out in the same way, only the solvent was changed from DMF to DMAc.

**Table S2.** Flow rates of poly(PFPA), amine A, and amine B solutions used for the PPM of poly(PFPA) in flow. The methodology was the same for 19F and 1H NMR controlled modifications.

| Ratio A/B | Flow rate poly(PFPA) / mL min^-1^ | Flow rate amine A / mL min^-1^ | Flow rate amine B / mL min^-1^ |
| --- | --- | --- | --- |
| 10/90 | 0.1 | 0.01 | 0.09 |
| 20/80 | 0.1 | 0.02 | 0.08 |
| 30/70 | 0.1 | 0.03 | 0.07 |
| 40/60 | 0.1 | 0.04 | 0.06 |
| 50/50 | 0.1 | 0.05 | 0.05 |
| 60/40 | 0.1 | 0.06 | 0.04 |
| 70/30 | 0.1 | 0.07 | 0.03 |
| 80/20 | 0.1 | 0.08 | 0.02 |
| 90/10 | 0.1 | 0.09 | 0.01 |

Characterization methods

NMR

Nuclear magnetic resonance spectra regarding the polymerization of PFPA were recorded using a Spinsolve 60 MHz NMR device from Magritek (Aachen, Germany) in a continuous flow mode. A PFA tubing was put in a glass guide tube and fed through the sample shaft of the device. A shimming routine is included immediately prior to the collection of each NMR spectrum to homogenise the magnetic field within the spectrometer, and ensure the spectra collected are of a high quality. Furthermore, by using 1/16’’ tubing to carry the product stream from reactor to spectrometer, the product stream is cooled to room temperature (because of the high surface area to volume ratio), which is essential to maintain a consistent inner temperature for the NMR magnet – again needed to yield high quality spectra.

Isolated polymers were analyzed using an Ascend 400 MHz spectrometer from Bruker (Billerica, Massachusetts, USA). Samples were dissolved in deuterated solvents and the number of scans was set to 64.

ATR FT-IR

Attenuated total reflection Fourier transform infrared spectra were recorded on a Vertex 80 from Bruker (Billerica, Massachusetts, USA) at 25 °C with a resolution of 2 cm^−1^. Each peak is assigned by its wavenumber and the intensity is indicated with *vs* (very strong), *s* (strong), *m* (medium), *w* (weak), or *vw* (very weak). Broad signals are indicated as “*br*”.

TGA

Thermal gravimetric analysis was carried out using a TGA 5500 from TA Instruments (New Castle, Delaware, USA) at a heating rate of 10.0 K min^−1^ under nitrogen atmosphere up to 1000 °C. Typically, about 5 mg material were weighed on a platinum pan for the measurements.

SEC

Size exclusion chromatography measurements were conducted using a Tosoh EcoSEC (Tokyo, Japan) SEC system equipped with a SDV 5 μm bead size guard column (50 × 8 mm) followed by three SDV 5 μm columns (300 × 7.5 mm, subsequently 100, 1000, and 10^5^ Å pore size). DMAc was used as eluent at 35 °C with a flow rate of 1.0 mL min^-1^. The SEC system was calibrated by using linear polymethyl methacrylate standards with the Mark-Houwink-Parameters K = 0.01298 mL g^-1^ and α = 0.688. Due to the inherent inaccuracy of SEC for materials that differ from the calibration polymer, molecular weights were rounded to the second digit.^[3]^

DSC

Differential scanning calorimetry was performed on a 214 Polyma DSC device from NETZSCH (Selb, Germany). Around 5 mg of sample were precisely (∆ = 0.005 mg) weighed in an aluminum pan with a pierced lid for measurement. An aluminum pan filled with air was used as a reference and the heating rate was typically set to 10 K min^-1^ for all measurements.

Results and Discussion

**Figure S3.** High-field ^1^H NMR spectrum of pentafluorophenyl acrylate (PFPA) in CDCl_3_.

**Figure S4.** High-field ^13^C NMR spectrum of pentafluorophenyl acrylate (PFPA) in CDCl_3_.

**Figure S5.** High-field ^19^F NMR spectrum of pentafluorophenyl acrylate (PFPA) in CDCl_3_.

**Figure S6.** FT-IR spectrum of pentafluorophenyl acrylate (PFPA). Mode: attenuated total reflection; resolution: 2 cm^-1^.

**Figure S7.** Temperature and residence time pairs investigated by the automation algorithm in the range of 70 to 120 °C and 10 to 60 minutes, respectively. Black squares: experiments chosen by Latin hypercube sampling for training of the algorithm. Red circles: Suggested experiments for the multi objective optimization of high monomer conversion and low dispersity. Blue circles: non-dominated solutions of the optimisation problem (Pareto front). Highlighted: most desirable reaction parameters: 94 °C and 56 minutes.


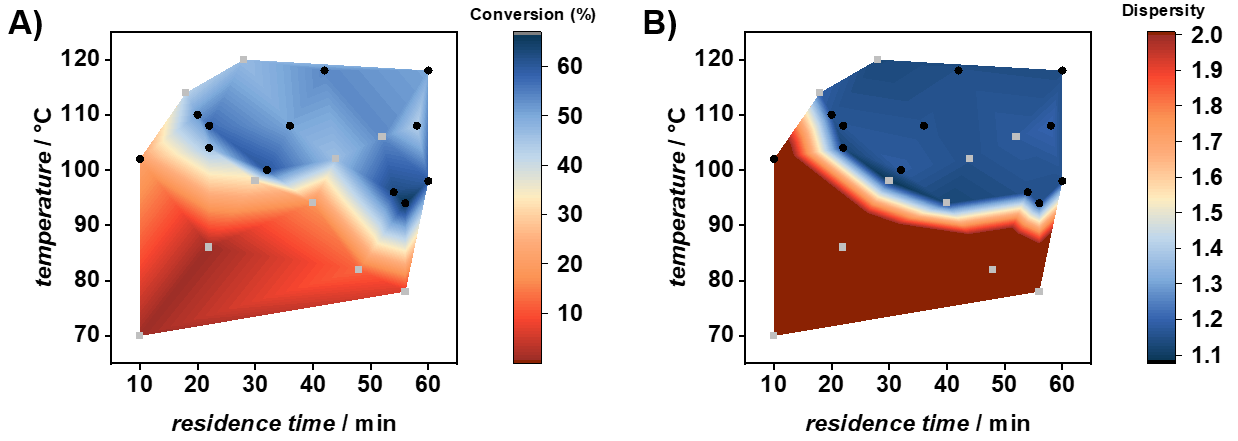


**Figure S8.** Conversion map (A) and dispersity map (B) of the automated optimization of the polymerization of PFPA in the investigated parameter space. Above 90 °C and 20 min residence time, the polymerization was found to run very controlled with reasonable monomer conversions. The loss of control over the polymerization mostly overlaps with the areas of low monomer conversion. Gray: LHC sampling experiments, black: algorithm-suggested optimization experiments.

**Figure S9.** Plot of calculated cumulative hypervolume vs. experiment number to visualize the hypervolume evolution during the optimization of the polymerization of PFPA.

**Figure S10.** Visualization of the hypervolume during the optimization process. Black squares: experiments chosen by Latin hypercube sampling for training of the algorithm. Red circles: Suggested experiments for the multi objective optimization. Blue circles: non-dominated solutions of the optimisation problem (Pareto front). The area created by the points on the Pareto front, the hypervolume, is an indicator of proximity to the utopian point, here: conversion = 1.0, *Đ* = 1.0. The hypervolume is examplatory visualized for only the right-most Pareto point. The dispersity of 2.0 was set as the upper limit to represent no control over the polymerization as in free radical polymerization. Measured dispersities exceeding this threshold were set to 2.0.

**Figure S11.** Low-field ^1^H NMR spectra of the reaction solutions before the polymerization (bottom), after polymerization (middle), and after amidation with benzylamine (top) without separating modification and polymerization. The highlighted area shows the vinyl double bond region. The double bonds were found to react with benzylamine in an Aza-Michael reaction, calling for separation of polymerization and modification of PFPA.

**Figure S12.** High-field ^19^F NMR spectrum of a mixture of 3,4,5-Trifluorobenzylamine and 4-(Trifluoromethyl)benzylamine in DMSO-*d*_6_. Aromatic and aliphatic fluorine atoms exhibit significantly different chemical shifts, allowing the calculation of the amount of the respective species by integration.

**Figure S13.** Stacked low-field 19F NMR spectra of poly(TFBAAm-*r*-TFMBAAm) modified with different rations of TFBA and TFMBA. No more signals in the region of aromatic poly(PFPA) resonances were found which confirms the quantitative conversion of PFP ester groups in 5 minutes at 70 °C. The singlet at -164.6 ppm can be attributed to the *para*-fluorine atom of TFBAAm, while the signals at -161.8, 165.5, and -172.0 ppm are assigned to detached pentafluorophenol species.

**Figure S14.** Offset normalized ^19^F NMR spectra of poly(TFBAAm-*r*-TFMBAAm) in the region between -55 and -140 ppm. The left peaks at -61 ppm are attributed to the CF_3_ group of TFMBAAm while the right peaks at -135 ppm are attributed to the *meta*-fluorine atoms of TFBAAm. The amount of TFMBA increased from green to red (10 – 90 mol%) while the amount of TFBA decreased from green to red (90 – 10 mol%), respectively.

**Figure S15.** Waterfall plot of the normalized ^1^H NMR spectra of poly(TFBAAm-*r*-TFMBAAm) in the region between 7.75 and 6.60 ppm recorded on a high-field device. A distinction between TFBAAm and TFMBAAm groups can be made, however no quantitative integration of characteristic signals was possible due to signal overlap. The amount of TFMBA increased from green to red (10 – 90 mol%) while the amount of TFBA decreased from green to red (90 – 10 mol%), respectively.

**Figure S16.** Linear fits of the ratio of TFBA against the peak absorbance of peaks A – G found in poly(TFBAAm-r-TFMBAAm). The peak absorbance of characteristic TFBA and TFMBA bond vibrations were found to correlate directly with the amount of respective group incorporated into the polymers.

**Figure S17.** SEC traces of poly(TFBAAm-*r*-TFMBAAm) in DMAc. A slight increase in molecular weight was observed with increasing amounts of heavier TFMBAAm in the polymer. The amount of TFMBA increased from green to red (10 – 90 mol%) while the amount of TFBA decreased from green to red (90 – 10 mol%), respectively. Calibration: PMMA.

**Figure S18.** *M*_n_ and *Đ* vs. TFBAAm content in poly(TFBAAm-*r*-TFMBAAm). The *M*_n_ (black) was found to slightly increase, while the dispersity (red) remained virtually unchanged.

**Figure S19.** Thermal decomposition profile of poly(TFBAAm-*r*-TFMBAAm) with different amounts of TFBAAm and TFMBAAm incorporated into the polymer. The amount of TFMBA increased from green to red (10 – 90 mol%) while the amount of TFBA decreased from green to red (90 – 10 mol%), respectively.

**Figure S20.** Offset DSC thermograms of poly(TFBAAm-*r*-TFMBAAm) from -30 to 70 °C. The glass transition temperatures (black lines) were found to be 20 °C, regardless of the modification ratio. The amount of TFMBA increased from green to red (10 – 90 mol%) while the amount of TFBA decreased from green to red (90 – 10 mol%), respectively.

**Figure S21.** High-field ^1^H NMR spectrum of a mixture of norbornenylmethylamine and benzylamine in DMSO-*d*_6_. Aromatic and double bond protons exhibit significantly different chemical shifts, allowing the calculation of the amount of the respective species by integration.

**Figure S22.** Offset normalized ^1^H NMR spectra of poly(NBMAAm-r-BAAm) in the region between 7.5 and 5.5 ppm. The left peaks at 7.25 ppm are attributed to the aromatic protons of BAAm while the right peaks at 6.0 ppm are attributed to the double bond protons of NBMAAm. The amount of NBMA increased from blue to red (10 – 90 mol%) while the amount of BA decreased from blue to red (90 – 10 mol%), respectively.

**Figure S23.** Linear fits of the ratio of NBMA against the peak absorbance of peaks A – D found in poly(NBMAAm-*r*-BAAm). The peak absorbance of characteristic NBMA and BA bond vibrations were found to correlate directly with the amount of respective group incorporated into the polymers.

**Figure S24.** SEC traces of poly(NBMAAm-*r*-BAAm) in DMAc. A slight increase in molecular weight was observed with increasing amounts of heavier NBMA in the polymer. The amount of NBMA increased from blue to red (10 – 90 mol%) while the amount of BA decreased from blue to red (90 – 10 mol%), respectively. Calibration: PMMA.

**Figure S25.** *M*_n_ and *Đ* vs. NBMAAm content in poly(NBMAAm-*r*-BAAm). The *M*_n_ (black) was found to slightly increase, while the dispersity (red) remained virtually unchanged.

**Figure S26.** Thermal decomposition profile of poly(NBMAAm-*r*-BAAm) with different amounts of NBMA and BA incorporated into the polymer. The amount of NBMA increased from blue to red (10 – 90 mol%) while the amount of BA decreased from blue to red (90 – 10 mol%), respectively.

**Table S3.** *T*_5%_ and *T*_95%_ temperatures of the thermal decomposition of poly(NBMAAm-*r*-BAAm) with different modification ratios.

| *Ratio NBMA/BA* | *T*_5%_ / °C | *T*_95%_ / °C |
| --- | --- | --- |
| 10/90 | 151 | 432 |
| 20/80 | 161 | 430 |
| 30/70 | 132 | 495 |
| 40/60 | 120 | 445 |
| 50/50 | 137 | 451 |
| 60/40 | 119 | 475 |
| 70/30 | 112 | 492 |
| 80/20 | 117 | 460 |
| 90/10 | 107 | 466 |

**Figure S27.** *T*_g_ of poly(NBMAA-*r*-BAAm) with different modification ratios. The *T*_g_ exhibits a minimum at a 50/50 modification ratio, which is believed to be caused by the hinderance of optimal chain orientation which causes less stable chain stacking.

Raw data

Raw data sets can be found in the RADAR4Chem Repository under the following digital object identifier: 10.22000/s2myp3gw533d4vq0.

# Author Contributions

A. P. G. proposed the original idea, performed the experiments, curated, and analyzed the data, and wrote the original draft (lead). S. T. K. and C. Y. P. W. helped with conducting the experiments, developed and adjusted the MATLAB code for the automation, and analyzed the data and corrected the original draft (lead). H. A. J. performed additional experiments on the automated platform and corrected the original draft (supporting). B. S. designed the 3D models of the 3D-printed flow reactor (lead) and helped with experiments and data analysis (supporting). O. P. helped with experiments and analysis of data (supporting). D. V. and C. W. S. supervised the project, validated the data, and corrected the paper (equal). D. V., N. J. W., and P. T. acquired funding (equal). N. J. W. and P. T. supervised the project and corrected the paper (lead).

[1] R. Bou Zerdan, Z. Geng, B. Narupai, Y. J. Diaz, M. W. Bates, D. S. Laitar, B. Souvagya, A. K. van Dyk, C. J. Hawker, *J. Polym. Sci.* **2020**, *58*, 1989.

[2] S. T. Knox, S. J. Parkinson, C. Y. P. Wilding, R. A. Bourne, N. J. Warren, *Polym. Chem.* **2022**, *13*, 1576.

[3] C. Y. P. Wilding, S. T. Knox, R. A. Bourne, N. J. Warren, *Macromolecules* **2023**.

[4] S. T. Knox, S. Parkinson, R. Stone, N. J. Warren, *Polym. Chem.* **2019**, *10*, 4774.
